# Impact of correlation of predictors on discrimination of risk models in development and external populations

**DOI:** 10.1186/s12874-017-0345-1

**Published:** 2017-04-19

**Authors:** Suman Kundu, Madhu Mazumdar, Bart Ferket

**Affiliations:** 1 Division of Cardiovascular Medicine, Vanderbilt University Medical Center, 2525 West End, Ste 300-A, Nashville, TN 37203 USA; 2 Department of Population Health Science and Policy, Institute for Healthcare Delivery Science, Icahn School of Medicine at Mount Sinai, New York, NY USA

**Keywords:** AUC, Correlation, External validation, Risk prediction, Simulation study

## Abstract

**Background:**

The area under the ROC curve (AUC) of risk models is known to be influenced by differences in case-mix and effect size of predictors. The impact of heterogeneity in correlation among predictors has however been under investigated. We sought to evaluate how correlation among predictors affects the AUC in development and external populations.

**Methods:**

We simulated hypothetical populations using two different methods based on means, standard deviations, and correlation of two continuous predictors. In the first approach, the distribution and correlation of predictors were assumed for the total population. In the second approach, these parameters were modeled conditional on disease status. In both approaches, multivariable logistic regression models were fitted to predict disease risk in individuals. Each risk model developed in a population was validated in the remaining populations to investigate external validity.

**Results:**

For both approaches, we observed that the magnitude of the AUC in the development and external populations depends on the correlation among predictors. Lower AUCs were estimated in scenarios of both strong positive and negative correlation, depending on the direction of predictor effects and the simulation method. However, when adjusted effect sizes of predictors were specified in the opposite directions, increasingly negative correlation consistently improved the AUC. AUCs in external validation populations were higher or lower than in the derivation cohort, even in the presence of similar predictor effects.

**Conclusions:**

Discrimination of risk prediction models should be assessed in various external populations with different correlation structures to make better inferences about model generalizability.

**Electronic supplementary material:**

The online version of this article (doi:10.1186/s12874-017-0345-1) contains supplementary material, which is available to authorized users.

## Background

Prediction models to estimate disease risk and identify individuals at high risk are widely advocated for optimizing prevention and management of multifactorial diseases. For several common complex diseases, including different forms of cancer, diabetes, and cardiovascular disease, many prediction models have been developed in various source populations [1–7]. The predictive performance of these risk models is typically assessed by evaluating discrimination. Discrimination is the ability of the model to separate those with and without events. After developing a risk model, it is essential to also investigate the model’s discriminative performance in external populations to judge the generalizability of the risk model. Because prediction models are developed to be used in new individuals, a risk model without appreciable predictive ability in an external population may have limited value for implementation in practice. Clinical practice guideline developers often systematically assess evidence on external validity before recommending prediction models. For example, performance of the Pooled Cohort Equations was evaluated first in two external cohorts and in more contemporary available data from the derivation cohorts, and then included in the 2013 ACC/AHA Guideline on the Assessment of Cardiovascular Risk [8].

It is often assumed that when a prediction model is validated within an external population, discriminative ability expressed by the area under the receiver operating characteristic curve (AUC) decreases [9]. However, sometimes the AUC increases, as observed in earlier validation studies [9–14]. Previous simulation studies have shown how the AUC is impacted by a different distribution of subject characteristics, including disease severity or occurrence (i.e., differences in “case-mix”) and heterogeneity in the effect sizes of risk factors among development and validation samples [15, 16]. These studies concluded that both differences in case-mix and predictor effects between derivation and validation populations must be assessed to fully appreciate the external validation results. When derivation and validation populations are similar regarding case-mix, external validation evaluates reproducibility of the prediction model. With an external validation procedure, one can determine whether the model suffered from ‘optimization bias’ by comparing its performance in the derivation and validation dataset. When case-mix differences are pronounced, external validation studies examine generalizability [17]. Demonstration of generalizability is more valuable, because it increases the likelihood that the prediction model will also perform well in new subjects. However, besides descriptive measures of predictors such as mean and standard deviation, correlation among the predictors may differ across populations. Thus, correlation of risk factors can be viewed as another dimension of case-mix, because it refers to the joint distribution of subject characteristics. Yet, it is not clear how different degrees of correlation might impact the AUC and how correlation should be interpreted along with other parameters that may change the AUC.

In this study, we first investigated the impact of correlation among predictors on the AUC in the development sample. Then we estimated the AUC when the developed risk models were applied in external populations with different correlation structures among the predictors. To put our findings into a more comprehensive context, we further explored how the distributions of predictors among cases and controls, and different strengths of predictive effects, can explain the variability of the AUC in external populations.

## Methods

We simulated several hypothetical populations with varying effect sizes and distribution of the predictors, as well as correlation among the predictors. We included correlation coefficients below 0.4 for the simulations, since these are typically observed for non-genetic predictors in biomedical research [18]. For each simulated population, we considered a binary disease outcome that can be predicted by two continuous predictors that follow Gaussian distributions. We used two approaches to construct the hypothetical populations of 100,000 individuals with a disease prevalence of 20%. This sample size was chosen to reduce uncertainty around the AUC estimates. We did not consider parameter uncertainty of predictor values and disease prevalence; thus, we did not report confidence intervals of AUCs. In both approaches, multivariable logistic regression models were fitted to predict disease risk in individuals. Each risk model developed in a population was validated in the remaining populations to investigate external validity.

### Approach I

In this approach, we drew random sets of predictor values from two normal distributions with predefined means and standard deviations, while using a correlation coefficient for the two predictors as defined for the total population [15, 19]. By fixing predefined independent beta coefficients for each predictor, we estimated the intercept term in the linear predictor (LP) of various fitted logistic regression models so that the average disease prevalence in the simulated data was 20%. Individual disease risks were subsequently estimated by transforming the linear predictors into predicted risks using the logit link function. Finally, we estimated binary disease status for each individual using the Bernoulli distribution. The different parameters used for each hypothetical population in this approach are presented as input parameters in Table 1.

**Table 1:** Input and estimated parameters in Approach I

Population	Input parameters			Estimated parameters					
	ρ	Normal (*μ, σ*)	Adjusted OR	Cases		Controls		SD of *β*_0_ *+* ∑*β*_*i*_ *X*_*i*_	AUC
				ρ	(*μ, σ*)	ρ	(*μ, σ*)		
A	0.2	*μ*: (0, 0); $\sigma$: (1, 1)	(1.5, 1.5)	0.17	*μ*: (0.37, 0.35); $\sigma$: (0.97, 0.97)	0.17	*μ*: (-0.09, -0.09); $\sigma$: (0.98, 0.98)	0.61	0.663
B	-0.1	,,	,,	-0.12	*μ*: (0.27, 0.28); $\sigma$: (0.98, 0.98)	-0.12	*μ*: (-0.07, -0.07); $\sigma$: (0.99, 0.99)	0.54	0.645
C	- 0.2	,,	,,	-0.22	*μ*: (0.26, 0.24); $\sigma$: (0.99, 0.99)	-0.22	*μ*: (-0.06, -0.06) $\sigma$: (0.99, 0.99)	0.51	0.639
D	0.1	,,	,,	0.07	*μ*: (0.33, 0.33) $\sigma$: (0.99, 0.99)	0.07	*μ*: (-0.08, -0.08) $\sigma$: (0.99, 0.99)	0.59	0.660
E	0.4	,,	,,	0.37	*μ*: (0.42, 0.42) $\sigma$: (0.97, 0.97)	0.37	*μ*: (-0.10, -0.10) $\sigma$: (0.98, 0.98)	0.67	0.676
F	0.2	,,	(1.5, 1.2)	0.18	*μ*: (0.34, 0.20) $\sigma$: (0.98, 0.98)	0.19	*μ*: (-0.08, -0.05) $\sigma$: (0.99, 0.99)	0.47	0.629
G	,,	,,	(1.2, 1.2)	0.19	*μ*: (0.16, 0.17) $\sigma$: (1, 1)	0.19	*μ*: (-0.04, -0.04) $\sigma$: (1, 1)	0.27	0.575
H	,,	,,	(1.5, 3)	0.10	*μ*: (0.41, 0.76) $\sigma$: (0.97, 0.97)	0.14	*μ*: (-0.10, -0.19) $\sigma$: (0.98, 0.98)	1.25	0.789
I	,,	,,	(0.8, 0.8)	0.20	*μ*: (-0.20, -0.20) $\sigma$: (1, 1)	0.19	*μ*: (0.05, 0.05) $\sigma$: (0.99, 0.99)	0.33	0.593
J	-0.1	,,	(1.5, 0.8)	-0.09	*μ*: (0.37, -0.20); $\sigma$: (0.99, 0.99)	-0.08	*μ*: (-0.08, 0.05); $\sigma$: (0.98, 0.98)	0.49	0.632
K	0.2	,,	,,	0.21	*μ*: (0.28, -0.11) $\sigma$: (0.99, 0.99)	0.21	*μ*: (-0.07, 0.03) $\sigma$: (0.99, 0.99)	0.42	0.616
L	0.4	,,	,,	0.40	*μ*: (0.24, -0.05); $\sigma$: (0.99, 0.99)	0.41	*μ*: (-0.06, 0.01); $\sigma$: (0.99, 0.99)	0.37	0.603
M	,,	Mean: (0, 0); SD: (1, 3)	(1.5, 1.5)	0.10	*μ*: (0.41, 2.47) $\sigma$: (0.97, 0.97)	0.14	*μ*: (-0.10, -0.62) $\sigma$: (0.98, 0.98)	1.37	0.804
N	- 0.2	,,	,,	-0.25	*μ*: (0.11, 2.25) $\sigma$: (1, 1)	-0.24	*μ*: (-0.03, -0.56) $\sigma$: (1, 1)	1.21	0.781
O	0.1	,,	,,	0.01	*μ*: (0.34, 2.38) $\sigma$: (0.98, 0.98)	0.04	*μ*: (-0.08, -0.59) $\sigma$: (0.99, 0.99)	1.31	0.795
P	0.4	,,	,,	0.30	*μ*: (0.56, 2.56) $\sigma$: (0.94, 0.94)	0.33	*μ*: (-0.14, -0.63) $\sigma$: (0.96, 0.96)	1.42	0.810

In each population, a disease prevalence of 20% was used

Population ‘A’ is considered as reference population; all other populations are compared w.r.t ‘A’

*SD* standard deviation, *OR* odds ratio

ρ: Pearson correlation between two continuous predictors

A risk factor X ~ Normal (*μ, σ*) implies ‘X’ follows a normal distribution with mean *μ* and variance *σ* ^*2*^

In Approach I, the adjusted ORs were pre-specified and thus considered as input parameters

Numbers are rounded to two decimals except for AUC estimates

### Approach II

In Approach II, instead of using common input parameters for the whole population, we used correlation coefficients, means, and standard deviations for the predictors stratified by cases and controls [20]. We then drew random sets of predictor values separately for cases and controls, and then combined them to construct the dataset of a hypothetical population. Unlike in Approach I, the independent or adjusted beta coefficient of the predictors was estimated by fitting a logistic model including both predictors. Thus, in Approach II, it is not possible to fix these beta coefficients in order to estimate the linear predictor. Again, in each population the proportion of cases was set to 20% of the total population size. The parameters used in Approach II are presented in Table 2.

**Table 2:** Input and estimated parameters in Approach II

Population	Input parameters for cases and controls		Estimated parameters for the population					
	ρ	Normal (*μ, σ*)	ρ	(*μ, σ*)	Unadjusted OR *	Adjusted OR **	SD of *β*_0_ *+* ∑*β*_*i*_ *X*_*i*_	AUC
A	Cases = 0.2 Ctrls = 0.2	*μ*Cases: (1, 2); Ctrls: (0, 0) $\sigma$Cases: (2, 2); Ctrls: (2, 2)	0.25	*μ*: (0.2, 0.4); $\sigma$: (2.04, 2.15)	1.28, 1.65	1.17, 1.60	1.13	0.770
B	Cases = 0.2 Ctrls = 0.4	,,	0.40	,,	,,	1.09, 1.60	1.09	0.765
C	Cases = 0.2 Ctrls = - 0.2	,,	-0.04	,,	,,	1.34, 1.68	1.25	0.785
D	Cases = 0.1 Ctrls = 0.1	,,	0.16	,,	,,	1.22, 1.62	1.17	0.777
E	Cases = - 0.1 Ctrls = - 0.1	,,	-0.02	,,	,,	1.35, 1.70	1.28	0.795
F	Cases = 0.2 Ctrls = 0.2	*μ*Cases: (1, 3); Ctrls: (0, 0) SD Cases: (2, 2); Ctrls: (2, 2)	0.27	*μ*: (0.2, 0.6); $\sigma$: (2.04, 2.33)	1.28, 2.12	1.11, 2.07	1.77	0.858
G	,,	*μ*Cases: (1, 3); Ctrls: (0, 2) SD Cases: (2, 2); Ctrls: (2, 2)	0.23	*μ*: (0.2, 0.2); $\sigma$: (2.04, 2.04)	1.28, 1.28	1.23, 1.23	0.67	0.676
H	,,	*μ*Cases: (1, 2); Ctrls: (0, 0) SD Cases: (2, 3); Ctrls: (2,3)	0.24	*μ*: (0.2, 0.4); $\sigma$: (2.04, 3.10)	1.28, 1.25	1.21, 1.22	0.80	0.705
I	,,	*μ*Cases: (1, 2); Ctrls: (0, 0) SD Cases: (2, 1); Ctrls: (2, 1)	0.27	*μ*: (0.2, 0.4); $\sigma$: (2.04, 1.28)	1.28, 7.39	1.05, 7.23	2.56	0.922

In each population, a disease prevalence of 20% was used

Population ‘A’ is considered as reference population and all other populations are compared w.r.t. ‘A’

SD: Standard Deviation; OR: Odds Ratio; Ctrls: controls

ρ: Pearson correlation between two continuous predictors

A risk factor X ~ Normal (*μ, σ*) implies ‘X’ follows a normal distribution with mean *μ* and variance $\sigma$ ^*2*^

*when a risk factor is normally distributed in both cases and controls and sigma is the common variance of the risk factor in both cases and controls, then unadjusted OR = exp((*μ*_*Case*_ *–μ*_*Control*_)/SD^2^) [19]

**adjusted ORs estimated by fitting logistic model

### Analyses

In both approaches, we assumed no measurement error and missing values of the predictors. We also assumed that there were no sources of bias and residual confounding, apart from the potential confounding effect between the two normally distributed predictors. We did not vary disease prevalence. Because the AUC statistic is calculated conditional on disease status, its value is theoretically independent of disease prevalence. We first alternately varied the correlation, mean, and standard deviation of the normal distributions, and the effect sizes of the predictors in Approach I to construct 16 hypothetical populations denoted by A-P. In Approach II, a presumed unadjusted effect size of the predictor was varied by increasing the difference in the mean values among cases and controls (i.e., absolute difference between *μ*_*Case*_ and *μ*_*Control*_). This process constructed 9 hypothetical populations denoted by A-I.

To explain possible changes in the estimated AUCs, we estimated the standard deviation (SD) of the resulting LP of each risk model in each development population. Higher variability of the LP indicates more heterogeneity of case-mix, which implies that individuals have a larger variety of characteristics, suggesting a higher AUC value [17]. For Approach I, we also reported the mean and SD of predictor values among cases and controls to observe the extent to which the two distributions were separated from each other. For Approach II, we reported the resulting mean and SD of predictor values in the total population. Further explanations and mathematical notations for each method are provided in the Supplemental Material.

All analyses were performed using R software (version 3.3.0; www.r-project.org). Simulation codes are available on request from the corresponding author.

## Results

### Model development

#### Approach I

**Fig. 1:**
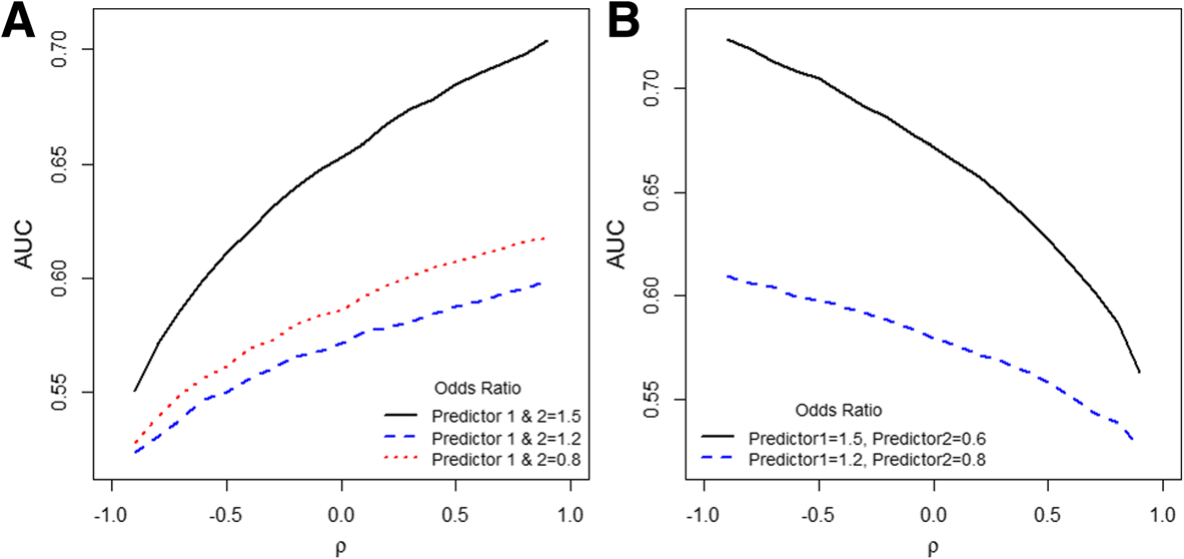
Relationships between AUC and correlation coefficient of two predictors: **a** Odds Ratios pointing in the same direction; **b** Odds Ratios pointing in opposite directions. Legend: Modeling is based on Approach I with populationμ : (0, 0);σ : (1, 1). ρ: Pearson correlation

a) When effects of the predictors pointed in the same direction (i.e. the ORs were both above 1), an increasingly positive correlation coefficient caused distributions of the predictors among cases and controls to be more separated from each other; thus, the SD of the LP increased. This in turn resulted in higher AUC values, while the mean and SD of the predictor distributions in the total population, and the adjusted ORs were kept fixed. For example, only correlation among predictors was varied in population A-E (Table 1) and the estimated AUC was lowest (0.64) in population C with a minimum correlation of -0.2. This gradually increased to 0.68 in population E, where the correlation was maximum (0.4). A similar trend is observed when the effects were made negative, suggesting ORs below 1 (Fig. 1a). However, when the effects of predictors pointed in opposite directions (i.e. one OR was above 1 and the other below 1), an opposite pattern was observed: more positive correlation yielded smaller SDs of the LP and lower AUC (Fig. 1b). For example, in population J-L (Table 1), the highest AUC was observed in population J where the correlation was minimal (-0.1). On the other hand, the lowest AUC appeared in population L, where the correlation was maximal. Figure 1 further illustrates this relation between AUC and correlation coefficient for the condition in which ORs point in the same direction and when they are in opposite direction. When effect sizes of predictors pointed in opposite directions, increasingly negative correlations consistently improved the AUC. The SD of the LP and the estimated AUC were perfectly related: when AUC was replaced by the SD of the LP for the y-axis of Fig. 1, an identical plot emerged (Additional file 1: Figure S1).

b) A higher SD of a predictor yielded a higher AUC when other parameters were kept fixed, as shown in Table 1 for populations ‘D’ and ‘O’. The SD of one predictor was increased from 1 to 3 and the AUC increased from 0.66 to 0.80.

c) As expected, when the effect size of a predictor in a risk model increased, the AUC increased. For example, the AUC in population F decreased to 0.63 from 0.66 as observed in population A, which had a higher OR of one predictor (Table 1).

#### Approach II

**Fig. 2:**
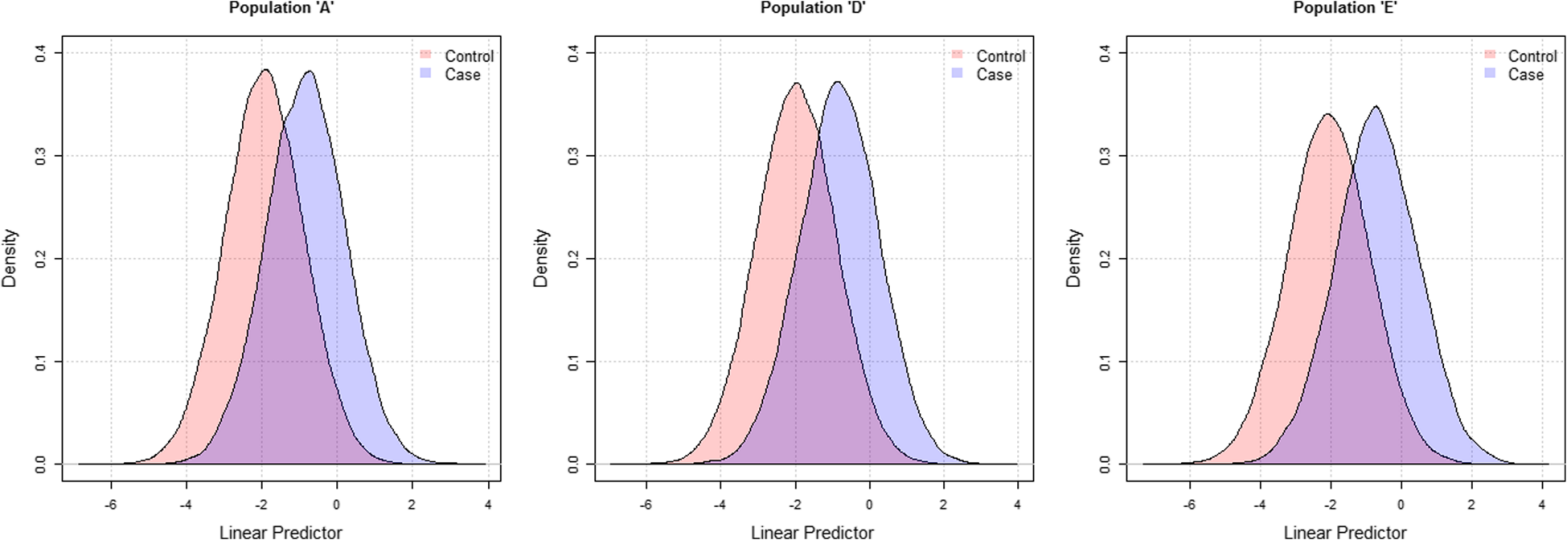
Amount of separation of linear predictor values for cases and controls in hypothetical populations with different AUCs. Legend: AUC of population ‘A’ is 0.770; ‘D’ is 0.777; and ‘E’ is 0.795. Modeling is based on Approach II with the following specifications: Population ‘A’: ρ_*Case*_ = 0.2, ρ_*Control*_ = 0.2; μ_*Cas*_ *e* : (1, 2); μ_*Control*_ : (0, 0); σ_*Cas*_ *e* : (2, 2); σ_*Control*_ : (2, 2). Population ‘D’: ρ_*Case*_ = 0.1, ρ_*Control*_ = 0.1; μ and σ same like in ‘A’. Population ‘D’: ρ_*Case*_ =0.1, ρ_*Control*_ =0.1; μand σ same like in ‘A’. Population ‘E’: ρ_*Case*_ =-0.1, ρ_*Control*_ = -0.1; μ and σ same like in ‘A’. Note: When the two linear predictor distributions are fully overlapping, for each chosen cut-off value on the range of linear predictor values, the proportion of false positives (controls labeled as high risk) equals true positives (cases labeled as high risk). This would result in an AUC of 0.5. Similarly when the two distributions are not overlapping, the AUC approximates 1

a) Table 2 shows that increasing the correlation of predictors among controls (and/or cases) implied less variation in the LP of risk models. This was indicated by a lower SD of the LP, which in turn resulted in a lower AUC value as shown in populations A-E. Figure 2 shows the extent of separation of linear predictor values in cases and controls, with higher separation observed for population E (AUC = 0.795) and lower for A (AUC = 0.770).

b) More separation of the distribution of cases and controls indicated a higher AUC value. For example, when the difference in the predictors’ means among cases and controls was larger (smaller), AUC increased (decreased), as observed in population A and F (A and G). Similarly, when the SD of a predictor among cases (or both cases and controls) increased, the amount of overlap between cases and controls increased, resulting in lower AUC values, as observed in populations A and H.

**Fig. 3:**
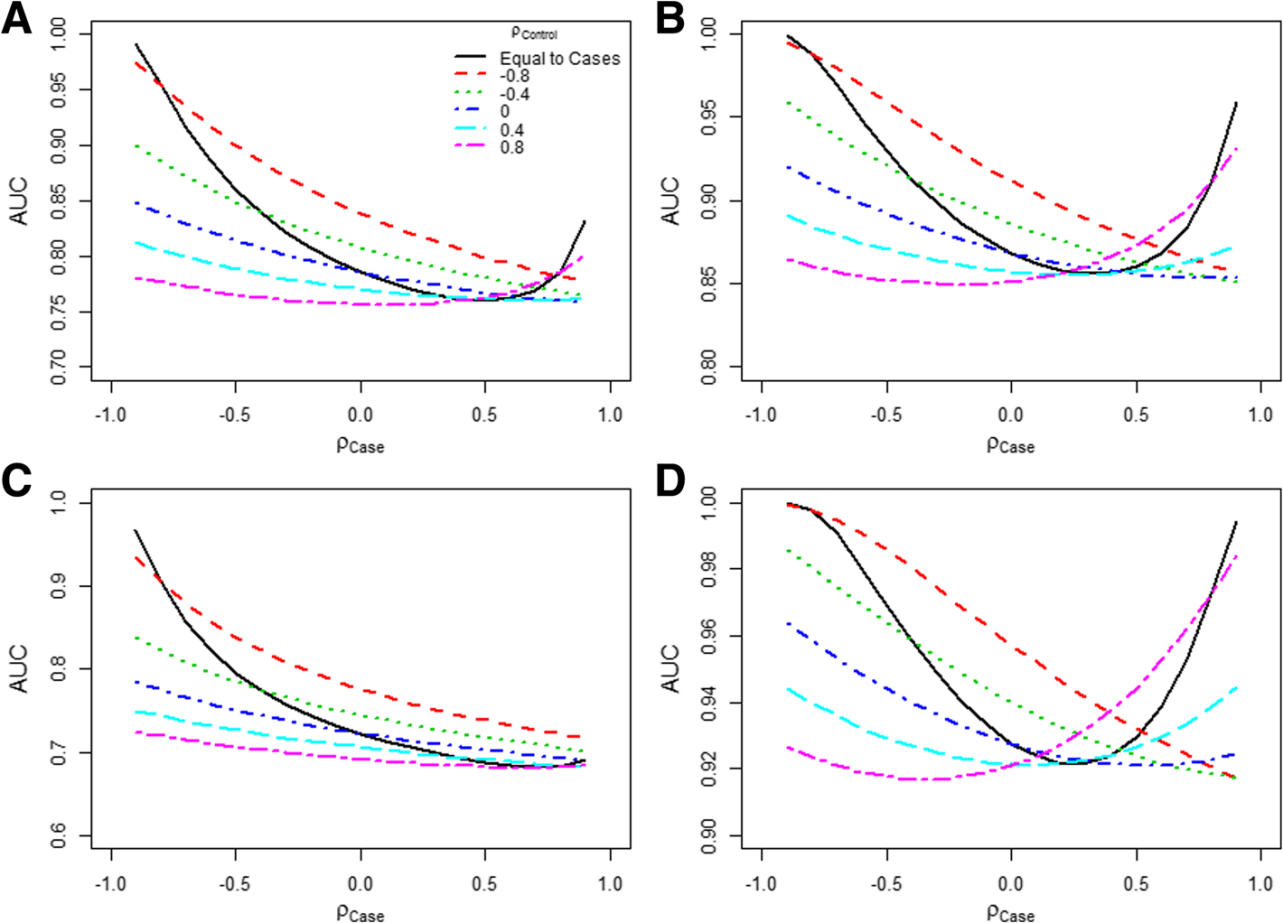
Relationships between AUC and fixed correlation coefficients in cases, while varying correlations of controls. Legend: **a** *μ*_*Case*_: (1, 2), *μ*_*Control*_: (0, 0), *σ*_*Case*_: (2, 2), *σ*_*Control*_: (2, 2). **b** *μ*_*Case*_: (1, 3), *μ*_*Control*_: (0, 0), *σ*_*Case*_: (2, 2), *σ*_*Control*_: (2, 2). **c** *μ*_*Case*_: (1, 2), *μ*_*Control*_: (0, 0), *σ*_*Case*_: (2, 3), *σ*_*Control*_: (2, 3). **d** *μ*_*Case*_: (1, 2), *μ*_*Control*_: (0, 0), *σ*_*Case*_: (2, 1), *σ*_*Control*_: (2, 1). ρ: Pearson correlation

Figure 3 shows the AUC as a function of correlation of cases, when correlation of controls was fixed at different levels. Four scenarios were considered with varying mean and SD values of cases and controls. In each scenario, increasingly negative correlations in cases lead to increasing improvement in AUC. The same results were observed with varying correlations of controls, while keeping the correlation of cases fixed at different levels (data not shown). With a very large positive correlation, the AUC also increased. However, when the correlation was positive but not very large, we did not observe a consistent pattern of change of AUC. As in Approach I, the SD of the LP and the estimated AUC were perfectly related, the plots in Fig. 3 and Additional file 1: Figure S2 appear very similar.

### Model validation

In both approaches, we observed the following results:

- The AUC of a risk model was highest when the model was validated in the same dataset (derivation sample) that was used to construct the model. Any risk model constructed in another population would perform equal to or less than the model fitted in the firstly mentioned derivation population (compare the rows in Tables 3 and 4). For example, in the first row of Tables 3 and 4, when risk models derived in different populations were validated in population A, the highest AUC was observed when the risk model was developed in population A.

**Table 3:** AUCs for risk models developed and validated in various populations: Approach I

Validated in population	Developed in population															
	A	B	C	D	E	F	G	H	I	J	K	L	M	N	0	P
A	***0.663***	*	*	*	*	0.656	0.663	0.652	0.663	0.556	**	**	*	*	*	*
B	0.645	***0.645***	*	*	*	0.632	0.645	0.631	0.644	0.534	**	**	*	*	*	*
C	0.639	*	***0.639***	*	*	0.626	0.639	0.622	0.639	0.534	**	**	*	*	*	*
D	0.660	*	*	***0.660***	*	0.649	0.660	0.649	0.659	0.545	**	**	*	*	*	*
E	0.676	*	*	*	***0.676***	0.670	0.676	0.670	0.676	0.570	**	**	*	*	*	*
F	0.624	*	*	*	*	***0.629***	0.624	0.602	0.625	0.578	**	**	*	*	*	*
G	0.575	*	*	*	*	0.571	***0.575***	0.570	0.575	0.526	**	**	*	*	*	*
H	0.770	*	*	*	*	0.728	0.770	***0.789***	0.767	0.502	**	**	*	*	*	*
I	0.593	*	*	*	*	0.590	0.593	0.587	***0.593***	0.534	**	**	*	*	*	*
J	0.531	*	*	*	*	0.529	0.530	0.531	0.530	***0.632***	**	**	*	*	*	*
K	0.540	*	*	*	*	0.571	0.540	0.502	0.543	0.615	***0.616***	**	*	*	*	*
L	0.542	*	*	*	*	0.541	0.540	0.541	0.542	0.602	**	***0.603***	*	*	*	*
M	0.804	*	*	*	*	0.792	0.804	0.800	0.804	0.693	**	**	***0.804***	*	*	*
N	0.781	*	*	*	*	0.759	0.781	0.775	0.781	0.692	**	**	*	***0.781***	*	*
**O**	0.795	*	*	*	*	0.782	0.795	0.790	0.795	0.686	**	**	*	*	***0.795***	*
P	0.810	*	*	*	*	0.802	0.810	0.806	0.810	0.695	**	**	*	*	*	***0.810***

*Risk models with the same adjusted ORs will have equal impact on an external validation population. Therefore, prediction models developed in population A-E and M-P will perform similarly in an external validation population, and thus the values indicated as ‘*’ in these columns are identical to those in column A

**The adjusted ORs in population J-K are the same and therefore perform similarly in an external validation population. Thus, the values indicated as ‘**’ in columns K and L are identical to those in column J

The numbers in bold indicate the AUC estimated in the development population

**Table 4:** AUCs for risk models developed and validated in various populations: Approach II

Validated in population		Developed in population								
		A	B	C	D	E	F	G	H	I
	**A**	***0.770***	0.768	0.767	0.770	0.767	0.767	0.753	0.754	0.762
	**B**	0.764	***0.765***	0.759	0.763	0.759	0.764	0.745	0.746	0.761
	**C**	0.783	0.777	***0.785***	0.785	0.784	0.773	0.774	0.774	0.763
	**D**	0.777	0.773	0.776	***0.777***	0.776	0.771	0.763	0.764	0.763
	**E**	0.790	0.781	0.795	0.793	***0.795***	0.777	0.785	0.786	0.763
	**F**	0.855	0.858	0.845	0.852	0.845	***0.858***	0.819	0.821	0.857
	**G**	0.664	0.655	0.672	0.667	0.671	0.651	***0.676***	0.676	0.642
	**H**	0.696	0.690	0.702	0.700	0.703	0.689	0.705	***0.705***	0.682
	**I**	0.896	0.914	0.864	0.885	0.863	0.917	0.811	0.814	***0.922***

The numbers in bold indicate the AUC estimated in the development population
- However, when a derived risk model was validated in different external populations, the AUCs could be higher or lower than the AUC in the derivation sample (compare values in any column in Tables 3 and 4). In other words, although the AUC in the derivation sample is not promising, the risk model can show a higher AUC in an external population. For example, in Table 3, the AUC of the risk model developed in population G is 0.575, but became as high as 0.810 when validated in population P. Conversely, it is also possible to develop a model with an apparently adequate AUC that performs poorly when validated on external populations. For example, in population H, the AUC was 0.789, which decreased to 0.587 when the same risk model was validated in population I (Table 3). Even when the adjusted ORs of the predictors were similar in both development and validation samples, higher AUC values could be obtained in the validation sample, as shown for the model derived in population A and validated in E (Table 3) and for the model derived in population G and validated in H (Table 4).

## Discussion

We constructed risk models in several hypothetical populations with varying correlations, standard deviations, and effect sizes among the predictors, and subsequently evaluated the performance of these models to investigate the impact of correlation on discriminative ability. Two approaches were used to construct hypothetical populations. In both approaches, the magnitude of the AUC in the development and external validation samples depended on the correlations among predictors.

There are some differences in the two approaches. In Approach I, the adjusted predictor effects were pre-specified and subsequently the correlation in the whole population was varied. In Approach II, the adjusted effects were a result of choosing the predictors’ distribution and correlation structure conditional on case and control status. To construct hypothetical populations, Approach II intuitively seems to be a more realistic approach than Approach I. In the latter, it is assumed that we know a priori the underlying independent effect of each predictor and that the degree of confounding, through correlated with the other predictor, varies across different populations. However, correlation coefficients of predictors can be very different for cases and controls [21, 22], which is difficult to include when using Approach I.

In the context of studying correlations as parameters independent of the predictors’ true effects, Approach I provides an interesting perspective. Using this approach, increasing positive correlations must result in less overlapping distributions of the LP among cases and controls. Similarly, increasing negative correlations result in more overlap, when the predictor effects point in the same direction. In this situation, mean predictor values among cases and controls must lie far apart (i.e., large unadjusted effects exist) when a large degree of confounding with positive correlation is introduced; mean values converge when confounding is removed with negative correlation. For the same reasons, less overlap results when the predictor effects point in the opposite direction and the correlation coefficient is made more negative.

In Approach II, the independent predictor effects are not known a priori, but result from varying the degree of confounding through the correlation. Unadjusted effects pointing in the same direction are created first, by specifying mean predictor values separately for cases and controls. By introducing more positive correlation in cases and controls combined (i.e. the correlation coefficient in the total population), adjusted effects will decrease. This results in significant overlap of LP distributions among cases and controls, especially when the differences in mean values of the predictors are small between cases and controls. However, when the correlation is equal among cases and controls, a correlation coefficient close to +1 will result in perfect discrimination: the AUC approximates 1 (Fig. 3). In that case, values of predictors will perfectly “move” in the same direction and the two distributions of the LP cannot be overlapping. This is especially the case when predictor means are further apart and standard deviations smaller (Fig. 3a, b and d).

Some of our results are in line with those of earlier studies. First, the AUC is generally highest in the population in which the risk prediction model is developed, since the coefficients of the model are best fitted to the data. Second, solely increasing the SD of predictors suggests higher variation in LP values or case-mix heterogeneity, and as a result, the model tends to discriminate better [15, 16]. As far as we know, only one previous study also evaluated the impact of correlation on the AUC, using Approach II only, and also showed that increasingly negative correlations improve the AUC [23]. However, this previous study only evaluated the effect on the AUC in the derivation sample.

The findings of our study should be interpreted in the light of some methodological considerations. First, even though discrimination in the form of the AUC is the most commonly used metric to investigate the predictive ability of risk models, we did not incorporate calibration and other performance measures [24, 25]. The potential merit of using risk models does not solely depend on their predictive performance, but also on their ability to improve treatment decisions and cost-effectiveness. Second, we only investigated logistic regression models, and did not consider interaction, collinear, and non-linear predictor effects. Third, we did not investigate non-Gaussian distributions of predictors. Fourth, we assigned disease status without considering differences in disease severity. Disease severity may vary with prevalence across different populations and generally changes the distribution of risk factor values. Therefore, disease prevalence may indirectly affect the AUC, also known as the spectrum effect [26–28]. We recommend investigating these potentially important issues in further research.

Our findings suggest that even when the AUC in the derivation sample is not promising, the same risk model can have a higher AUC at external validation [9, 14]. Conversely, even though the AUC in both derivation and a particular validation dataset is high, the same risk model can perform poorly in another external population. As shown in Approach I, when the adjusted predictor effects are similar across derivation and validation cohorts, the underlying mechanism for the variation in AUCs can be explained by heterogeneous correlations among populations. When the AUCs and one or more adjusted predictor effects are different, other factors may play a role, including: i) underlying independent predictor effects may vary, or ii) predictors and/or disease status were misclassified or measured differently. Varied underlying predictor effects can occur due to heterogeneity in (ignored or overlooked) effects such as interactions, non-linearity, associations with residual confounders, and disease biology. As demonstrated in Approach II, when unadjusted effects are similar across the derivation and validation samples, stronger correlations in the validation sample may lead to smaller adjusted effects, less heterogeneity in the LP, and a lower AUC.

Recently, a method was proposed to investigate the relatedness of development and validation samples [17]. It uses a model including the envisioned predictors and disease status as covariables to predict membership of an underlying source population for individuals in the derivation and validation samples. If membership can be accurately predicted, the derivation and validation populations are considered not similar in terms of subject characteristics and outcome status. However, this method requires that both the derivation and the validation datasets are at hand, which is rare. Usually, prediction models are externally validated using the modeling equations provided in the published literature.

## Conclusions

We demonstrated using two different approaches illustrating that description of the mean, SD, effect sizes, and correlations among predictors can provide important information about differences in AUCs across development and external populations. Although some of these metrics are reported in predictive modeling studies, reporting of the correlation structure among predictors is rare. Therefore, we call for more detailed reporting of summary statistics, in addition to emphasizing the need for validation of models in various independent populations to ensure generalizability. The latter will guarantee quicker incorporation within clinical practice guidelines and increase accuracy of clinical decision making.

## Additional file

Additional file 1: Mathematical details for simulating hypothetical populations, Figure S1, and Figure S2. (DOCX 102 kb)

## Abbreviations

AUC: Aarea under the receiver operating characteristic curve
LP: Linear predictor
OR: Odds ratio
SD: standard deviation
