# Supplementary material for: Impact of correlation of predictors on discrimination of risk models in development and external populations
Source: BMC Med Res Methodol. 2017 Apr 19;17:63. doi: 10.1186/s12874-017-0345-1 (PMC5395845; doi:10.1186/s12874-017-0345-1)
Supplement: Additional file 1: — Mathematical details for simulating hypothetical populations, Figure S1, and Figure S2. (DOCX 102 kb) [file 12874_2017_345_MOESM1_ESM.docx]

**Supplemental Material**

**Mathematical details for simulating hypothetical populations**

**A. Approach I**

The method constructs a dataset with two continuous predictors that follow Gaussian distributions and estimates of disease status for hypothetical populations. It requires specification of input parameters of population disease prevalence, mean, SD, the Pearson product-moment correlation coefficient, and odds ratios (ORs) for the two continuous predictors. The dataset is constructed using a simulation procedure that involves the following three steps:

1. **Modeling predictors:** A random set of 100,000 values is selected from a multivariate normal distribution with means ($\mu_{i}$) and SDs ($\sigma_{i}$) of the two predictors (X_i_) and the correlation ($\rho$), such that X_i_ ~ N($\mu_{i},\sigma_{i}$); i=1, 2 and correlation (X_1_, X_2_)= $\rho$
2. **Modeling individual disease risks:** Disease risks are calculated from the logistic regression equation:

*Logit (risk_j_) =* = *Linear predictor (LP_j_)*

where: risk*_j_* = disease risk for individual *j; j=1 to 100,000*

$\beta_{0}$= intercept,

$\beta_{i}$= *log(OR_i_)*, with *OR_i_* being the Odds Ratio of the predictor i*,*

When $\beta_{i}$and $X_{i}$are known, $\beta_{0}$ is obtained by solving the logistic regression equation such that the average risk in the hypothetical population is equal to the specified population disease risk.

1. **Modeling disease status:** Disease status (0 or 1) is assigned to each individual with the probability of developing disease (1) being equal to *risk_j._*

**B. Approach II**

Approach II, requires specification of the mean and SD for each continuous predictor, and correlation coefficients separately for cases and controls. The individually generated ‘case’ and ‘control’ populations are then combined to construct the hypothetical population. The method consists of two steps:

1. **Modeling predictors:** Predictor values are generated separately for cases and controls. To generate a population of size 100,000 with 20% disease prevalence, a random set of 20,000 cases was selected from a multivariate normal distribution with means () and SDs () of the two predictors (X_i-case_) and the correlation ($\rho_{Case}$) such that X_i-case_ ~ N(); i=1, 2 and correlation (X_1-case_, X_2-case_)= $\rho_{Case}$. Similarly, a random set of 80,000 controls was selected from the specified mean, SD, and correlation for controls.
2. **Constructing populations:** Combine cases and controls to construct a hypothetical population.

Population=$\left[\begin{matrix} \Pr edictor-1 & \Pr edictor-2 & Diseasestatus \\ \text{X}_{_{1}\text{-case}}\text{ } & \text{X}_{\text{2-case}} & \text{Cases (1)} \\ \text{X}_{_{1}\text{-control}} & \text{X}_{\text{2-control}} & \text{Control (0)} \end{matrix}\right]$

**Supplementary Figure 1:** Relationship between standard deviation of the linear predictor and correlation coefficient of two predictors in Approach I: A) Odds Ratios point in the same direction, B) Odds Ratios point in opposite directions.


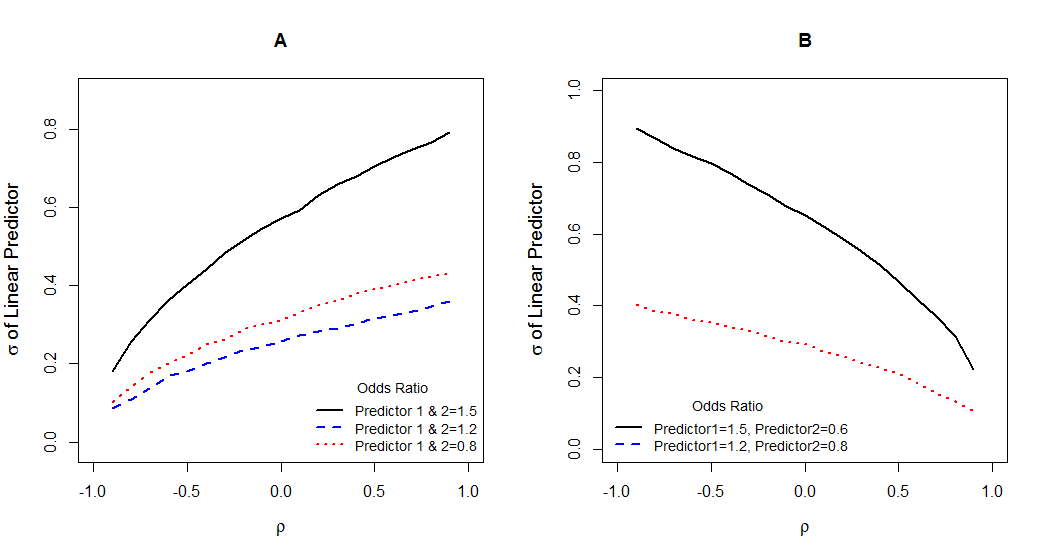


The modeling is based on Approach I with population$\mu$: (0, 0);$\sigma$: (1, 1)

$\rho$: Pearson correlation

**Supplementary Figure 2:** Relationship between standard deviation of the linear predictor and correlation in cases in Approach II


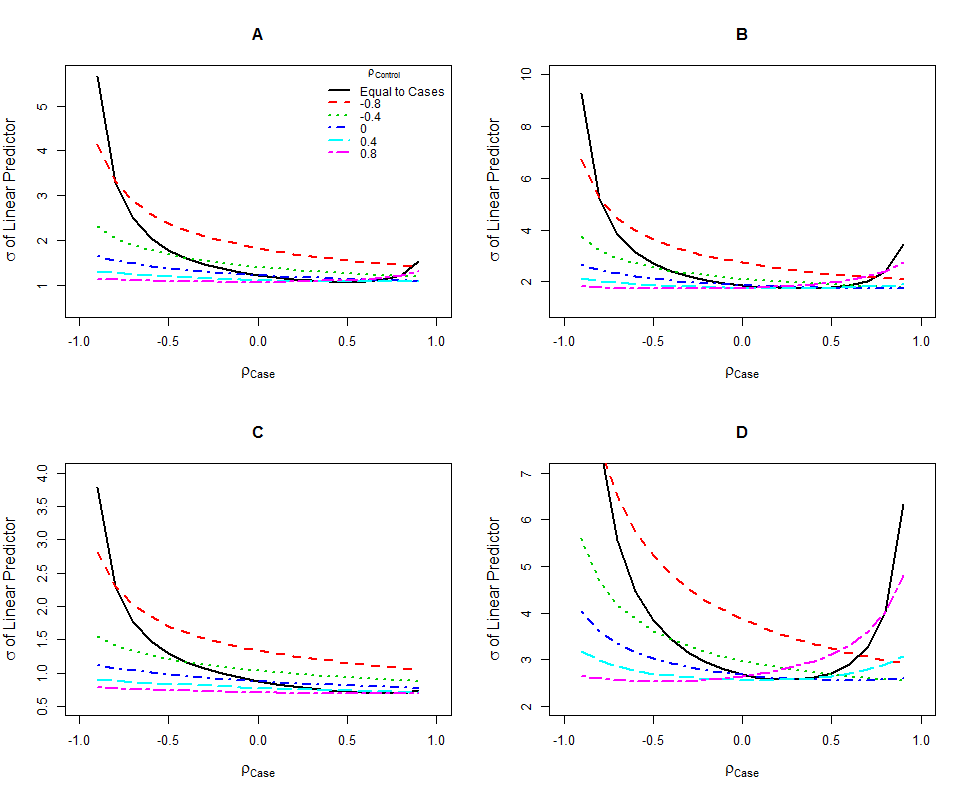


Figure A: $\mu_{Case}$: (1, 2), $\mu_{Control}$: (0, 0), $\sigma_{Case}$: (2, 2), $\sigma_{Control}$: (2, 2)

Figure B: $\mu_{Case}$: (1, 3), $\mu_{Control}$: (0, 0), $\sigma_{Case}$: (2, 2), $\sigma_{Control}$: (2, 2)

Figure C: $\mu_{Case}$: (1, 2), $\mu_{Control}$: (0, 0), $\sigma_{Case}$: (2, 3), $\sigma_{Control}$: (2, 3)

Figure D: $\mu_{Case}$: (1, 2), $\mu_{Control}$: (0, 0), $\sigma_{Case}$: (2, 1), $\sigma_{Control}$: (2, 1)

$\rho$: Pearson correlation
